# Shikonin Inhibits Tumor Growth in Mice by Suppressing Pyruvate Kinase M2-mediated Aerobic Glycolysis

**DOI:** 10.1038/s41598-018-31615-y

**Published:** 2018-09-28

**Authors:** Xiaoyue Zhao, Yanan Zhu, Jianhua Hu, Longwei Jiang, Limin Li, Shaochang Jia, Ke Zen

**Affiliations:** 1 Bayi Clinical Medicine School, Nanjing University of Chinese Medicine, No. 34, Yanggongjing Street, Nanjing, Jiangsu 210002 China; 2 State Key Laboratory of Pharmaceutical Biotechnology, Jiangsu Engineering Research Center for MicroRNA Biology and Biotechnology, Nanjing University School of Life Sciences, Nanjing, Jiangsu 210093 China

## Abstract

Shift metabolism profile from mitochondrial oxidative phosphorylation to aerobic glycolysis (Warburg effect) is a key for tumor cell growth and metastasis. Therefore, suppressing the tumor aerobic glycolysis shows a great promise in anti-tumor therapy. In the present study, we study the role of shikonin, a naphthoquinone isolated from the traditional Chinese medicine Lithospermum, in inhibiting tumor aerobic glycolysis and thus tumor growth. We found that shikonin dose-dependently inhibited glucose uptake and lactate production in Lewis lung carcinoma (LLC) and B16 melanoma cells, confirming the inhibitory effect of shikonin on tumor aerobic glycolysis. Treatment of shikonin also decreased tumor cell ATP production. Furthermore, pyruvate kinase M2 (PKM2) inhibitor or activator respectively altered the effect of shikonin on tumor cell aerobic glycolysis, suggesting that suppression of cell aerobic glycolysis by shikonin is through decreasing PKM2 activity. Western blot analysis confirmed that shikonin treatment reduced tumor cell PKM2 phosphorylation though did not reduce total cellular PKM2 level. *In vitro* assay also showed that shikonin treatment significantly promoted tumor cell apoptosis compared to untreated control cells. Finally, when mice implanted with B16 cells were administered with shikonin or control vehicle, only shikonin treatment significantly decreased B16 tumor cell growth. In conclusion, this study demonstrates that shikonin inhibits tumor growth in mice by suppressing PKM2-mediated aerobic glycolysis.

## Introduction

Compared to normal non-proliferating cells, tumor cells display a high aerobic glycolysis (Warburg effect). In fact, metabolic switch from oxidative phosphorylation to aerobic glycolysis is a major feature of tumor cell and a key for tumor cell maintaining rapid growth and metastasis^1–4^. As the final rate-limiting enzyme of cell glycolysis, pyruvate kinase M2 (PKM2) plays a critical role in tumor cell metabolic switch from oxidative phosphorylation to aerobic glycolysis^5–7^. Therefore, reagents that can suppressive aerobic glycolysis particularly modulating PKM2 activity have shown a great potential in developing anti-tumor drug^8^.

Shikonin is a natural product isolated from the roots of the Chinese herbs Lithospermum erythrorhizon, Arnebia euchroma and Onosma paniculata^9–11^. Previous studies demonstrate that shikonin has a broad therapeutic effects ranging from anti-inflammatory, anti-oxidant, anti-cancer, wound healing to anti-microbial^12–14^. Recently shikonin has been shown to kill certain cancer cells and inhibit the migration and invasion of cancer cells^15^ through a number of possible mechanisms, including the inhibition of protein tyrosine kinase (PTK)^16^, the activities of DNA topoisomerases^17^, and tumor necrosis factor receptor-associated protein 1 (TRAP1) expression^18^. Other mechanisms involved in shikonin-induced cancer cell death include upregulation of p53^19^. However, the exact mechanism by which shikonin inhibits tumor cell proliferation, migration and invasion remains incompletely understood. It is not clear whether shikonin can be used as an effective anti-cancer reagent *in vitro* and *in vivo*.

In the present study, we tested the effect of shikonin on the proliferation and apoptosis of various cancer cells *in vitro* and *in vivo*. Our results show that shikonin dose-dependently inhibits tumor cell aerobic glycolysis and growth while promotes tumor cell apoptosis. The mechanistic study also suggests that the mechanism underlying the anti-cancer effect of shikonin is to inhibit the phosphorylation of PKM2 and thus suppress PKM2-switched tumor cell aerobic glycolysis.

## Results

### Shikonin suppresses tumor cell proliferation

**Figure 1:**
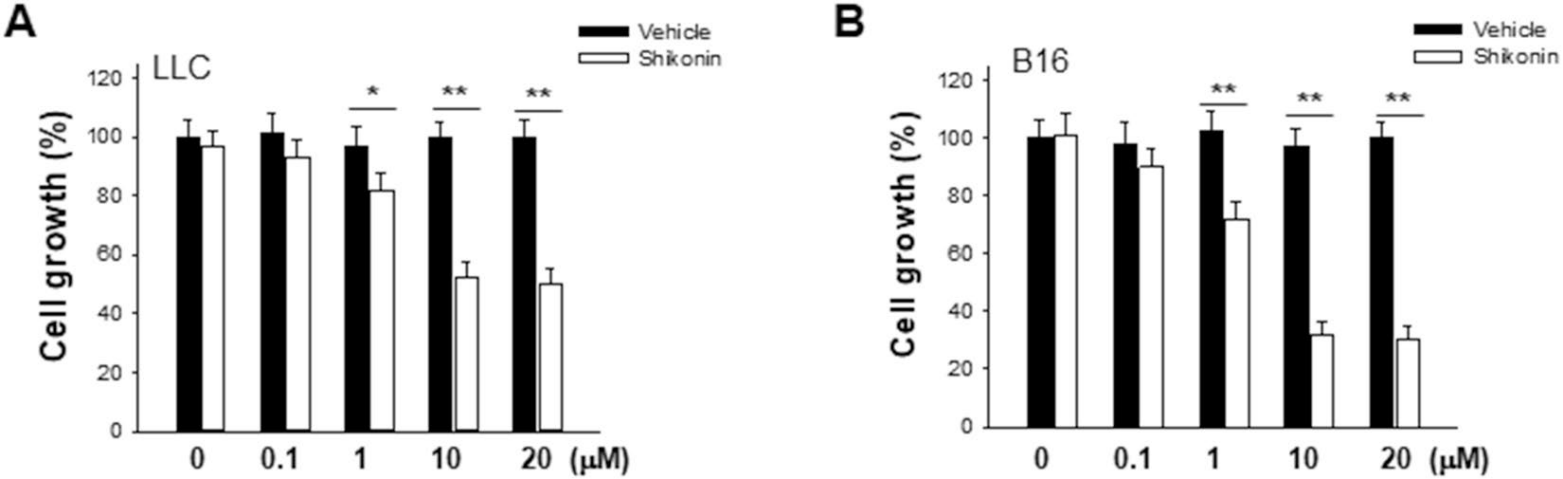
Shikonin inhibits tumor cell proliferation. LLC (**A**) and B16 (**B**) tumor cells were treated with various concentration of shikonin or vehicle (DMSO) for 24 h. Data were presented as a means ± SD of 3–4 individual experiments with three samples in each time point. *P < 0.05. **P < 0.01.

Previous study in our laboratory has demonstrated that shikonin could reduce exocytosis process in tumor cells^20^, an event closely related to tumor cell aerobic glycolysis. To test the effect of shikonin on tumor cell growth, we assessed the proliferation of LLC and B16 tumor cells in the presence of various concentration of shikonin. Shikonin was dissolved in DMSO and DMSO served as vehicle control. As shown in Fig. 1, after 24 h treatment, shikonin dose-dependently inhibited both LLC and B16 tumor cell growth, suggesting that shikonin may affect a common signaling pathway of tumor proliferation.

### Shikonin inhibits tumor cell aerobic glycolysis

**Figure 2:**
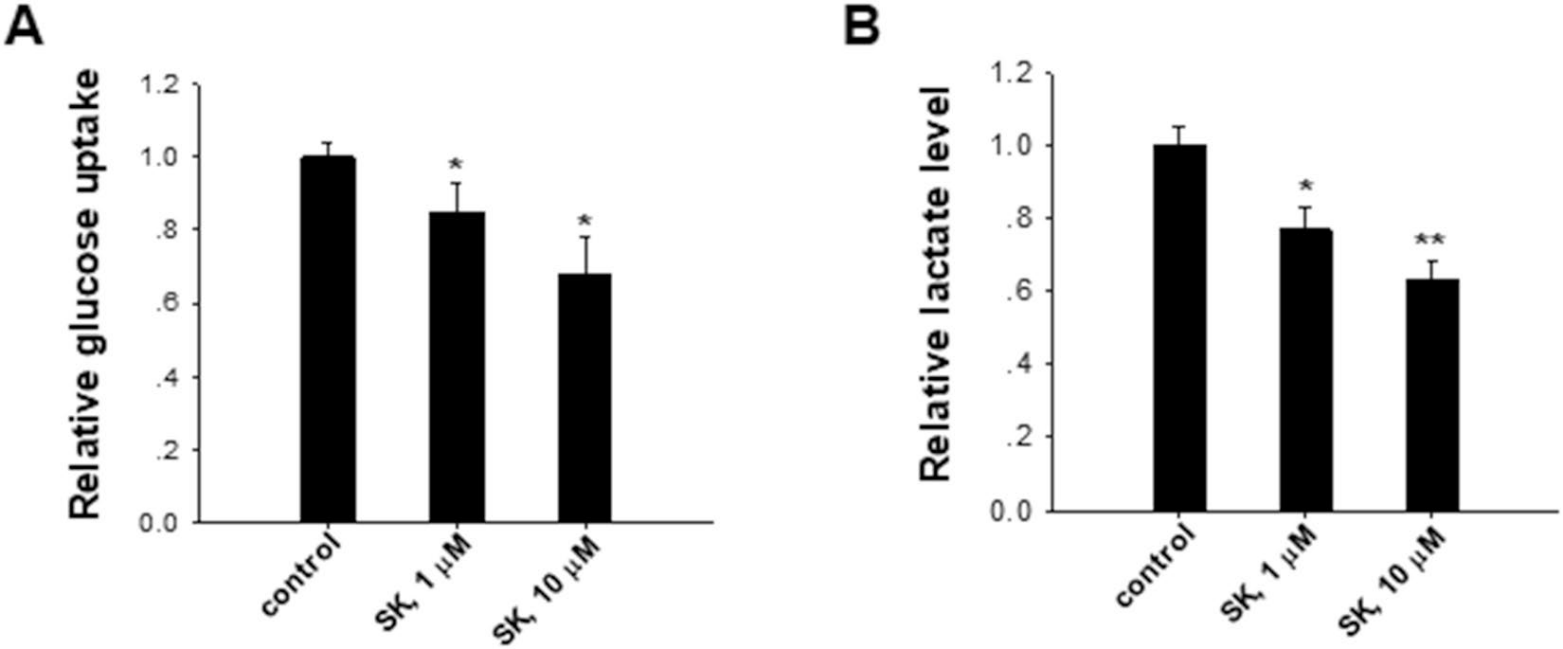
Shikonin suppresses tumor cell aerobic glycolysis. (**A**) Shikonin (SK) decreased relative glucose uptake in B16 cells in a dose-dependent manner. (**B**) Shikonin (SK) reduced relative lactate production in B16 cells in a dose-dependent manner. Data were presented as a means ± SD of 3–4 individual experiments with three samples under each condition. *P < 0.05. **P < 0.01.

**Figure 3:**
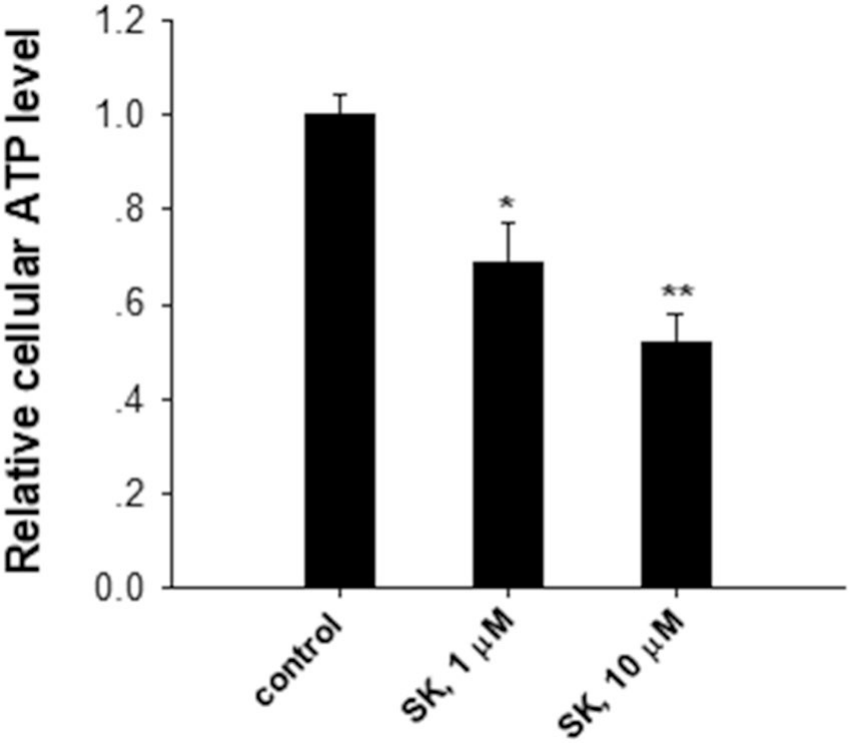
Shikonin decreases ATP level in B16 cells in a dose-dependent manner. Data were presented as a means ± SD of three individual experiments with three samples under each condition. *P < 0.05. **P < 0.01.

Compared to normal cells, rapid proliferating tumor cells switch their metabolism from oxidative phosphorylation to aerobic glycolysis (Warburg effect)^21–23^. Given that aerobic glycolysis is a key for most tumor cells to maintain rapid growth and metastasis, we tested whether shikonin inhibited tumor cell growth via blocking the aerobic glycolysis in tumor cells. In this experiment, we assessed lactate production and glucose uptake in B16 cells with or without shikonin treatment. As shown in Fig. 2, shikonin dose-dependently reduced the glucose uptake and lactate production in B16 cells, suggesting that shikonin suppressed tumor cell aerobic glycolysis. This observation is in agreement with previous findings of shikonin as a Warburg effect inhibitor^2,24^. In agreement with that shikonin treatment suppressed tumor cell aerobic glycolysis, the ATP level in B16 cells was decreased by shikonin in a dose-dependent manner (Fig. 3).

### Shikonin inhibits cell aerobic glycolysis via decreasing PKM2 phosphorylation

**Figure 4:**
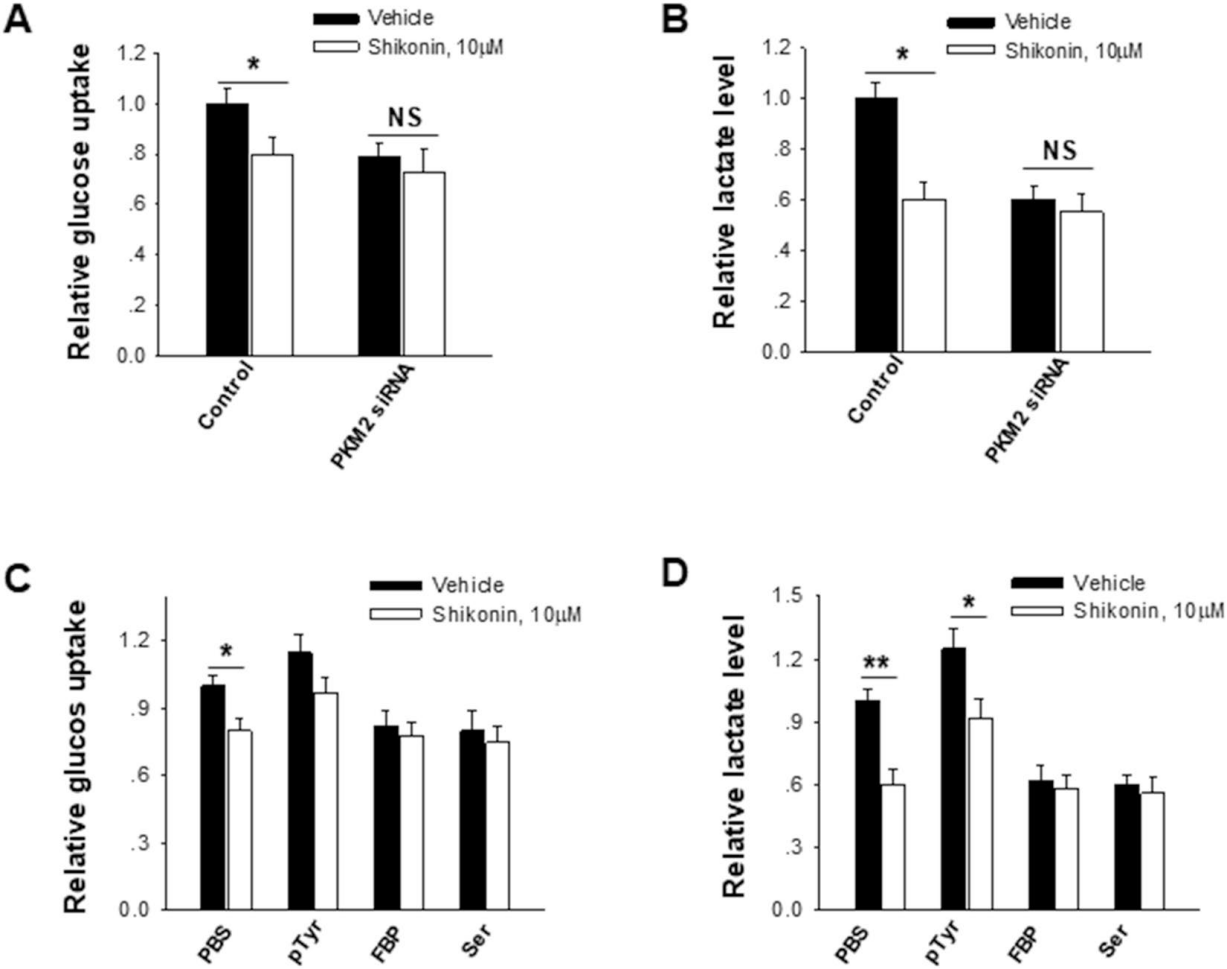
Effect of shikonin on suppressing tumor cell aerobic glycolysis is dependent on PKM2. (**A**) Knockdown of PKM2 in B16 cells via PKM2 siRNA abolished the effect of shikonin on tumor cell glucose uptake. (**B**) Knockdown of PKM2 in B16 cells abolished the effect of shikonin on tumor cell lactate production. (**C**) Modulation of PKM2 activity affected the relative glucose uptake in B16 cells. (**D**) Modulation of PKM2 activity affected the relative lactate production in B16 cells. Data were presented as a means ± SD of three individual experiments with three samples under each condition. *P < 0.05.

Tumor cell aerobic glycolysis is controlled by various factors, including glycolysis substrates and metabolic rate-limiting enzymes. As the final rate-limiting enzyme, PKM2 has been shown to play a critical role in switching tumor cell metabolism from oxidative phosphorylation to aerobic glycolysis^3,4,25^. To test whether PKM2 is involved in the inhibitory effect of shikonin on tumor cell aerobic glycolysis, we transfected tumor cells with PKM2 siRNA to knock down PKM2 level and then treated tumor cells with shikonin. As shown in Fig. 4A,B, control cells treated with 10 nM shikonin displayed a significantly reduced glucose uptake and lactate production. In contrast, tumor cells treated with PKM2 siRNA showed no significant difference of glucose uptake and lactate production before and after shikonin treatment. This result implies that shikonin may execute its function through altering PKM2 expression. It has been known that PKM2 activity can be modulated by pTyr, a phosphotyrosine peptide that can specifically phosphorylate PKM2 and promote PKM2 dimeric formation^4^, as well as fructose (1,6) bisphosphate (FBP) and serine (Ser), which arrest PKM2 in their active tetramer confirmation^3^. We also treated tumor cells with pTyr, FBP or Ser in the presence or absence of shikonin. The results showed that control cells (treated with PBS solution) displayed a lower glucose uptake and lactate production following 10 μM shikonin treatment, whereas no significant effect of shikonin on tumor cell aerobic glycolysis was detected after modulating PKM2 activity with pTyr, FBP or Ser (Fig. 4C,D). These results are in agreement with the notion that shikonin inhibits tumor cell aerobic glycolysis through affecting PKM2 expression or activity.

**Figure 5:**
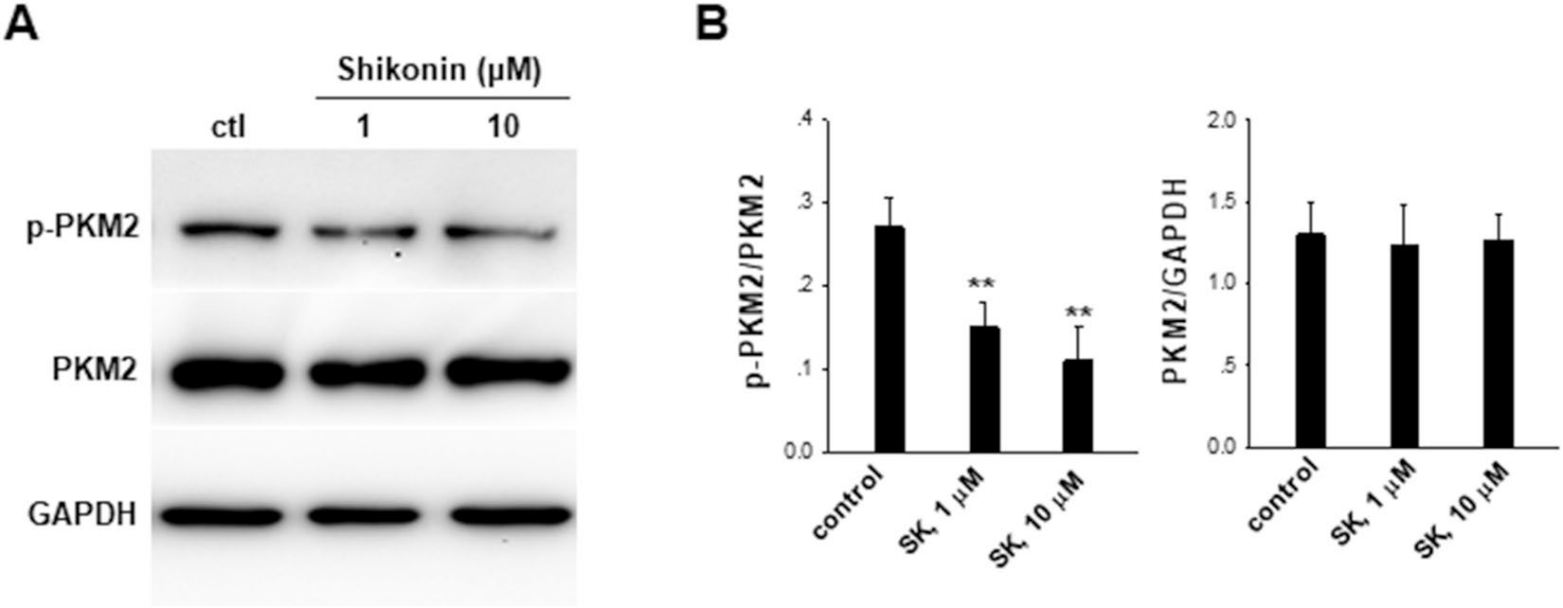
Shikonin (SK) treatment decreases phosphorylation of PKM2 (p-PKM2). (**A**) Representative western blot images of p-PKM2, PKM2 and GAPDH in B16 cells treated with or without shikonin. The raw WB data of Fig. 5A were shown in Supplementary Material. (**B**) Analysis results of western blot images in panel A. Data were presented as a means ± SD of three individual experiments. *P < 0.05. **P < 0.01.

To test this, we tested the protein expression level and phosphorylation of PKM2 in B16 cells with or without shikonin treatment by western blot analysis. As shown in Fig. 5A,B, shikonin treatment dose-dependently reduced the PKM2 phosphorylation in B16 cells although it did not affect the tumor cell PKM2 protein level. The result suggests that shikonin treatment may affect the PKM2 activity instead of expression level.

### Shikonin promotes tumor cell apoptosis

**Figure 6:**
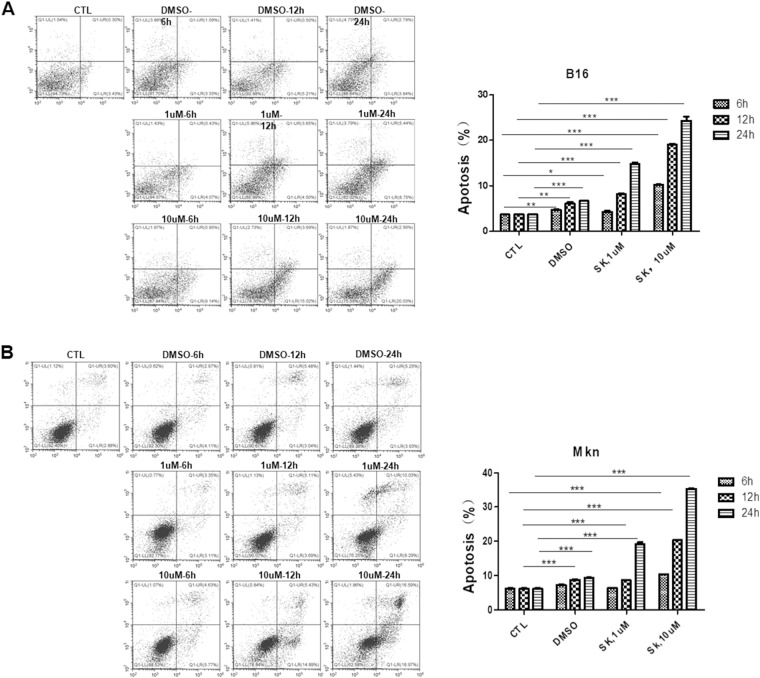
Shikonin treatment increases tumor cell apoptosis in a dose-dependent and time-dependent manner. (**A**) Left panel: representative flow cytometry image of B16 cell apoptosis labeled with FITC-Annexin V/PI. Right panel: analysis results of flow cytometry images. (**B**) Left panel: representative flow cytometry image of gastric cancer cell apoptosis labeled with FITC-Annexin V/PI. Right panel: analysis results of flow cytometry images. Data were presented as a means ± SD of three individual experiments in triplicate. *P < 0.05. **P < 0.01. ***P < 0.001.

Given that aerobic glycolysis plays a critical role in tumor cell survival^22,23^, we next examined the potential effect of shikonin on tumor cells apoptosis using flow cytometry. In this experiment, B16 cells and gastric cancer MKN-45 cells were treated with 0, 1 or 10 μM shikonin at different time points, respectively. Cells were then labeled with fluorescently Annexin V–FITC and propidium iodide (PI) for measuring early and late apoptosis of tumor cells^24^. As shown in Fig. 6, shikonin treatment increased apoptosis of two different tumor cells in both dose-dependent and time-dependent manner.

### Shikonin suppresses tumor cell growth in mouse model

**Figure 7:**
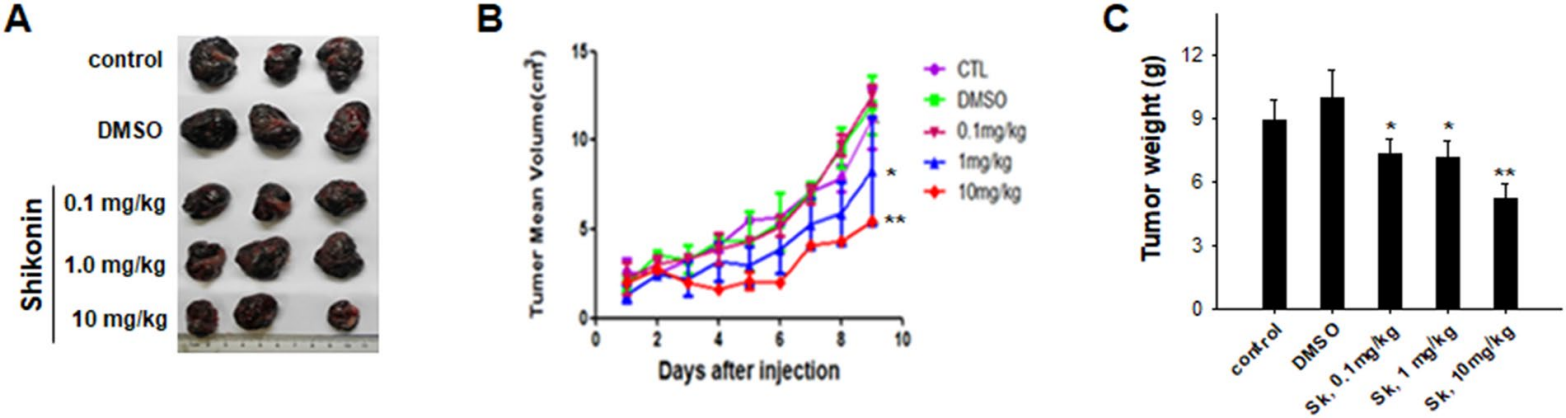
Shikonin treatment inhibits the growth of implanted tumor in SCID mice. (**A**) Representative tumor images in SCID mice implanted with B16 melanoma. (**B**) B16 tumor size in mice on various days following injection with various doses of shikonin. (**C**) B16 tumor weight in mice on day 9 after treatment with various doses of shikonin. Data were presented as a means ± SD (n = 6). **P < 0.01.

To test the effect of shikonin on tumor growth *in vivo*, we performed experiments using 6-week-old male SCID mice. In the experiment, B16 melanoma cells were injected subcutaneously into SCID mice (1 × 10^6^ cells per mouse, 6 mice per group). After the xenografts were established, the tumor-bearing mice were administered with shikonin (0, 0.1, 1 and 10 mg/kg, respectively) via intraperitoneal injection. As shown in Fig. 7, shikonin treatment inhibited B16 cell growth in SCID mice in a dose-dependent manner compared to PBS (control) and DMSO treatment. A significant reduction of tumor size (Fig. 7B) and weight (Fig. 7C) was observed when shikonin was injected at concentration of 1 or 10 mg/kg.

## Discussion

In the present study, we demonstrate that shikonin can inhibit tumor proliferation *in vitro* and *in vivo* through decreasing PKM2-mediated aerobic glycolysis switch in tumor cells. This study provides shikonin as an effective anti-cancer drug candidate.

In recent years, accumulating evidences demonstrate that metabolic switch from oxidative phosphorylation to aerobic glycolysis (Warburg effect) is critical for tumor cells maintaining high proliferation and metastasis^21–23^. Blockade of tumor cell aerobic glycolysis particularly the PKM2-mediated aerobic glycolysis switch thus shows a great potential in anti-cancer therapy. Employing cell and mouse model, we have characterized the inhibitory effect of shikonin on tumor cell proliferation, as well as the possible mechanism under such event. Several pieces of evidence support that shikonin inhibits tumor proliferation through decreasing PKM2-mediated aerobic glycolysis switch. Firstly, shikonin reduced the proliferation of LLC and B16 tumor cells and this effect was correlated with its inhibitory effect on tumor cell aerobic glycolysis, Secondly, the effect of shikonin on suppressing tumor cell aerobic glycolysis could be offset by modulating PKM2 level and activity. As shown in Fig. 4, PKM2 knockdown in tumor cells via PKM2 siRNA or modulation of PKM2 activity by pTyr, FBP or serine largely abolished the inhibition of tumor cell aerobic glycolysis by shikonin. Finally, western blot analysis directly showed that shikonin treatment decreased the phosphorylation of PKM2 in B16 cells though did not affect the total cellular PKM2 level.

Although our results demonstrate that shikonin suppresses tumor cell aerobic glycolysis via inhibiting PKM2 phosphorylation, the molecular basis of reduction of PKM2 phosphorylation by shikonin remains unknown at this stage. Through studying the activity of PKM2 after treating PKM2 with different small molecules, previous studies have shown that PKM2 Activator II (DASA), as well as glycolytic intermediates FBP and serine, can modulate PKM2 activity through arresting PKM2 in tetramer structural form^26,27^. It may be true that shikonin affects PKM2 activity in a similar manner. In addition, as protein kinase Akt2 has been reported to be able to promote PKM2 phosphorylation in tumor cells^28,29^, shikonin may inhibit PKM2 phosphorylation through suppressing the expression and activity of such protein kinase. Given that PKM2 phosphorylation may switch the conformation of PKM2 from tetramer to dimer, inhibition of PKM2 phosphorylation by shikonin may have a similar role in preventing PKM2 tetramer-to-dimer conformation switch.

## Materials and Methods

### Animal model

6-week-old male severe combined immune deficiency (SCID) mice (nu/nu) were obtained from the Model Animal Research Center of Nanjing University (Nanjing, China) and maintained under specific pathogen-free conditions at Nanjing University. The experiments on mice were approved by Institutional Animal Care and Use Committee, Nanjing University, and all experiments were performed in accordance with relevant guidelines and regulations. B16 melanoma cells were injected subcutaneously into SCID mice (10^6^ cells per mouse, 6 mice per group). After the xenografts were established, the tumor-bearing mice were administered with shikonin (0, 0.1, 1, 10 mg/kg) via intraperitoneal injection. Shikonin was purchased from Sigma-Aldrich (St. Louis, MO, USA) and dissolved in DMSO (Sigma-Aldrich). The length, width and height of the tumors were measured with digital calipers every day and tumor volume was calculated accordingly^30^. On the ninth day, the mice were sacrificed and the tumors were weighed.

### Cell culture

B16 cells and gastric cancer MKN-45 cells were obtained from Shanghai Institute of Cell Biology, Chinese Academy of Sciences (Shanghai, China) and maintained in RPMI 1640 medium (Gibco, NY) supplemented with 10% fetal bovine serum (FBS) and 1% penicillin–streptomycin within a humidified atmosphere containing 5% CO_2_ at 37 °C. Cells using for functional and mechanism studies in this study were tested and authenticated using short tandem repeat (STR) method by Shanghai Institute of Cell Biology. For evaluating the effect of shikonin on the viability and metabolic status of B16 cells, different concentration of shikonin were added into B16 cell culture medium and cells were incubated for 24 h.

### Cell proliferation

Cell proliferation was assayed by WST (water-soluble tetrazolium salt) assay using Cell Counting Kit-8 (Sigma-Aldrich) according to the manufacturer’s instructions^31^. Briefly, LLC and B16 cells were seeded into 96-well plates (Corning) at a density of 10^4^ cells per well in DMEM and incubated for 24 h (37 °C and 5% CO_2_). The medium was then replaced with either serum-free DMEM or serum-free DMEM containing various concentrations (0, 0.01, 0.1, 1 or 10 µM) of shikonin (the total volume in each well was 200 µl). After incubation for another 24 h, the number of viable cells was determined by measurement of the absorbance (OD450 nm).

### Apoptosis assay

Apoptosis of cells was detected using an Annexin V–FITC/propidium iodide (PI) staining assay. Flow cytometric analysis of apoptotic cells was carried out using an Annexin V–FITC/PI staining kit (Invitrogen). After washes with cold PBS, the cells were re-suspended in binding buffer (100 mM HEPES, 100 mM NaCl, and 25 mM CaCl_2_, pH 7.4) and stained with Annexin V-FITC/PI at room temperature in darkness for 15 min. Apoptotic cells were then evaluated by gating PI and Annexin V–positive cells on an FACSCalibur (BD Biosciences). All experiments were performed in triplicate.

### Western blot

Cellular proteins were extracted as described previously^20,24^. Antibodies against PKM2, p-PKM2 purchased from Abcam (Shanghai, China) were used for western blotting. GAPDH (Cell Signaling Technology, CA) served as an internal control.

### Measurement of lactate production, glucose uptake and ATP production

The lactate level in the cell culture medium was measured with lactate assay kit (#K607-100, BioVision, Milpitas, CA, USA) according to the method described previously^32^. Glucose in cell lysates was measured with glucose assay kit (BioVision, #K606-100). For detecting glucose uptake and lactate production, the culture supernatants of tumor cells with different treatments were collected and the fresh culture media was used as control. Equal amounts (2–10 µl) of samples were added to a 96-well plate and the volume of each well was then adjusted to 50 µl with Glucose or Lactate Assay Buffer. Meantime, a standard curve was prepared with the same protocol. After 30 min reaction at 37 °C in dark, the absorbance (OD570 nm) or fluorescence intensity (Ex/Em = 535/590 nm) were measured. The uptake of glucose was determined by subtracting the glucose level in tested samples from the initial glucose level in fresh media. The production of lactate was determined referring standard curve. Considering the cell number of individual sample may be different, all the levels of glucose or production of lactate were finally normalized to the protein level. ATP levels were measured using an ATP assay kit (Celltiter-Glo Luminescent Cell Viability Assay, Promega).

### Statistical analysis

Each experiment was representative of at least three independent experiments. The data were presented as the means ± SD of at least three independent experiments. Differences between groups were analyzed using Student’s *t*-test and the differences were considered to be statistically significant at *P* < 0.05.

## Electronic supplementary material

Raw WB data (Fig. 5A)
