# Supplementary material for: Shikonin Inhibits Tumor Growth in Mice by Suppressing Pyruvate Kinase M2-mediated Aerobic Glycolysis
Source: Sci Rep. 2018 Sep 28;8:14517. doi: 10.1038/s41598-018-31615-y (PMC6162216; doi:10.1038/s41598-018-31615-y)
Supplement: Supplementary file 1 — Raw WB data (Fig. 5A) [file 41598_2018_31615_MOESM1_ESM.pdf]

Shikonin Inhibits Tumor Growth in Mice by Suppressing Pyruvate Kinase M2-mediated Aerobic Glycolysis

Xiaoyue Zhao[1†], Yanan Zhu[2†], Jianhua Hu[1], Longwei Jiang[1], Limin Li[2]*, Shaochang Jia[1]*, and Ke Zen[2]*

[1]Bayi Clinical Medicine School, Nanjing University of Chinese Medicine, No. 34, Yanggongjing Street, Nanjing Jiangsu 210002; [2]State Key Laboratory of Pharmaceutical Biotechnology, Jiangsu Engineering Research Center for MicroRNA Biology and Biotechnology, Nanjing University School of Life Sciences, Nanjing, Jiangsu 210093, China

Supplementary Figure 1; Raw WB data for Fig. 5A.

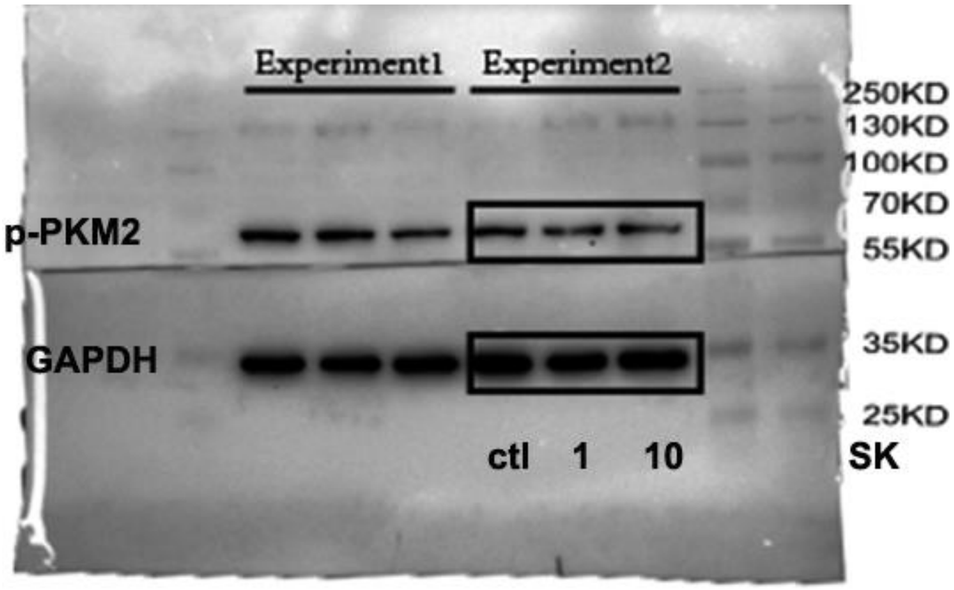
Experiment1
Experiment2
p-PKM2
GAPDH
250KD
130KD
100KD
70KD
55KD
35KD
25KD
ctl
1
10
SK

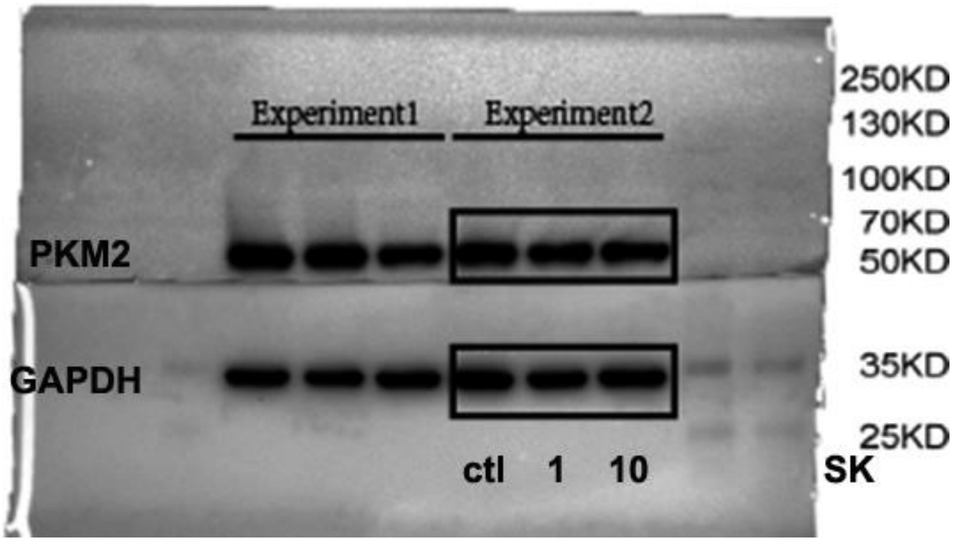
Experiment1
Experiment2
PKM2
GAPDH
250KD
130KD
100KD
70KD
50KD
35KD
25KD
ctl
1
10
SK
